# The Role of Muscle Strength in the Sit-to-Stand Task in Parkinson's Disease

**DOI:** 10.1155/2023/5016802

**Published:** 2023-10-23

**Authors:** José Fidel Baizabal-Carvallo, Marlene Alonso-Juarez, Robert Fekete

**Affiliations:** ^1^Department of Sciences and Engineering, University of Guanajuato, León, Mexico; ^2^Instituto Politécnico Nacional, Mexico City, Mexico; ^3^New York Medical College, Valhalla, New York, NY, USA

## Abstract

**Background:**

Rising from a chair or the sit-to-stand (STS) task is frequently impaired in individuals with Parkinson's disease (PD). These patients commonly attribute such difficulties to weakness in the lower extremities. However, the role of muscle strength in the STS transfer task has not been fully elucidated.

**Objective:**

We aim at determining the role of muscle strength in the STS task.

**Methods:**

We studied 90 consecutive patients with PD and 52 sex- and age-matched controls. Lower limb strength was determined in both legs by clinical examination using the Medical Research Council Scale, dynamometric (leg flexion) and weighting machine (leg pressure) measures. Patients were interrogated regarding the presence of subjective lower limb weakness or allied sensations.

**Results:**

There were 20 patients (22.2%) with abnormal STS task (item 3.9 of the MDS-UPDRS-III ≥2 points). These patients had higher modified Hoehn and Yahr stage (*P*  <  0.001) and higher total motor scores of the MDS-UPDRS(*P*  <  0.001), compared with 70 PD patients with normal STS task. Patients with abnormal STS task endorsed lower limb weakness more frequently and had lower muscle strength in the proximal lower extremities, compared to PD patients with normal STS task and normal controls. The presence of perceived lower limb weakness increased the risk of an abnormal STS task, OR: 11.93 (95% C.I. 1.51–94.32), whereas a hip extension strength ≤9 kg/pressure also increased the risk of abnormal STS task, OR: 4.45 (95% C.I. 1.49–13.23). In the multivariate regression analysis, bradykinesia and decreased hip strength were related to abnormal STS task.

**Conclusions:**

Patients with PD and abnormal STS task complain more commonly of lower limb weakness and have decreased proximal lower limb strength compared to patients with PD and normal STS task, likely contributing to abnormalities in performing the STS task.

## 1. Introduction

Parkinson's disease (PD) is a neurological disorder characterized by degeneration of dopaminergic cells in the substantia nigra [1]. Patients with PD develop diverse motor and nonmotor manifestations during the course of the disease. Bradykinesia, muscle rigidity, and tremor are the most commonly recognized motor manifestations; however, other symptoms, such as difficulty in rising from a chair or performing the sit-to-stand (STS) task are frequently reported by these patients [2]. The STS task plays an important role in general mobility determining functional independence and influencing the quality of life [2, 3]. It is estimated that about 44% of patients with PD have some degree of difficulty to perform this task [2].

Abnormalities in kinematics, joint torques, and postural instability have been deemed important in the pathogenesis of abnormal STS task in patients with PD [4–6]. Patients with PD frequently complain of decreased muscle strength in the lower limbs and attribute their difficulties for rising to the chair to such perceived weakness [7, 8]. Clinicians frequently face the clinical paradox of finding normal muscle strength in the lower limbs of patients with PD during the neurological examination, while observing prominent abnormalities in the STS task attributed to weakness in a proportion of cases.

A number of studies have shown decreased muscle strength in patients with PD by means of reproducible, quantitative methods [9–11]. This includes studies reporting decreased lower limb muscle strength, mainly at the hip level, associated with difficulties for STS task [5, 12]. Contrasting research studies have not found differences in muscle strength during the STS task in PD patients compared with healthy controls [13]. However, the number of individuals enrolled in these studies is relatively small, for that reason, we studied muscle strength in patients with PD and matched healthy controls by clinical and dynamometric methods and correlated with the STS task in these patients.

## 2. Materials and Methods

We enrolled consecutive patients diagnosed with PD from a tertiary-care center for movement disorders. Inclusion criteria considered patients of both sexes, age equal or older than 18 years and with a diagnosis of Parkinson's disease, according to the Queen Square Brain Bank Criteria [14]. Exclusion/elimination criteria considered patients with Parkinsonism who would not fulfill diagnostic criteria for PD and the presence of musculoskeletal or neurological abnormalities that may result in decreased motor strength in the lower limbs independently of PD.

The clinical evaluation was performed by the Movement Disorders Society Unified Parkinson's Disease Rating Scale part III or motor score (MDS-UPDRS-III). We assessed for signs of severe neuropathy and spasticity, including abnormal muscle reflexes and the Babinski sign in all patients. Item 3.9 “arising from chair” was used to assess for the STS task. The item has five subcategories as follows: (0) no problems, patient is able to arise quickly without hesitation; (1) slight: patient is slower than normal and may need more than one attempt or move forward in the chair to arise; (2) mild: patient pushes him/herself up from the arms of the chair without difficulty; (3) moderate: patient needs to push off, but tends to fall back, or may have to try more than once using the arms of the chair, but can get up without help; and (4) severe: patient is unable to arise without help. For the purpose of this study, we defined an “abnormal” item 3.9 “arising from chair” as a score equal or higher than 2, as a score of 1 may just reflect hesitation or poor preparation to stand up from the chair, and it is uncertain if this represents a clear abnormality. We calculated composite scores for leg tremor, rigidity, and bradykinesia by totaling items of each leg and dividing them between two.

The disease stage was determined by the modified Hoehn and Yahr scale [15]. The total levodopa equivalent daily dose (LEDD) at the time of the clinical evaluations was also assessed [16]. We recorded the presence of dyskinesia during the neurological examination. We also evaluated the presence of orthostatic hypotension (OH) while seated and 3 minutes after standing and defined OH as sitting-to-standing systolic blood pressure drop ≥20 mmHg or a diastolic blood pressure drop ≥10 mmHg [17].

We assessed the frequency of perceived lower limb weakness in all patients and matched controls. Abnormal lower limb sensations, such as “heavy” or “fatigued” legs, were considered within the spectrum of lower limb weakness. These sensations were rated by each patient from 1 (very mild) to 10 (very severe). Patients also rated the functional impact of weakness and allied sensations as “mild”: limitation only for long hikes, i.e., more than 5 blocks; “moderate”: limitation to walk between 1 and 5 blocks; and “severe”: limitation to walk few meters or to walk at all. In addition, we enrolled 52 sex- and age-matched healthy controls. We assessed lower limb strength, perceived weakness, and allied sensations in these controls.

The strengths of iliopsoas, rectus femoris, and sartorius and pectineus muscles were assessed by manual muscle testing using the Medical Research Council (MRC) Scale [18]] and by dynamometric calculation (ZP-500, Digital Force Gauge, Alipo, China). The rectus femoris, vastus lateralis, vastus medialis, and vastus intermedius were assessed during manual test of leg (knee) extension. The hamstrings (biceps femoris, semitendinosus, and semimembranosus), gracilis, sartorius, gastrocnemius, and popliteus muscles strength were individually assessed in each leg during sequential leg pressure over the floor using a calibrated weighing machine (BF-679W, Tanita, Arlington Heights, Illinois, USA). Reproducibility for these tests has been previously determined [8]. Leg pressure requires maximal muscle strength (force) generated by rapid muscle contractions (velocity). Muscle power (force × velocity) has been found decreased in PD and such deficit becomes more evident at increasing velocities of isokinetic muscle contractions [19]. The study was approved by the local committee of ethics at the Sante Medical Tower (number: 20220501) and patients and controls provided written informed consent to participate in the study.

## 3. Statistics

We summarized data in means and standard deviations and percentages. The *X*^2^ and Fisher's exact test were used to compare nominal or ordinal data. The paired *t*-test was used to compare means between patients and age-matched controls. Risks were calculated with odd ratios (ORs) and 95% confidence intervals (C.Is). Statistical evaluations were performed using SPSS version 22; a *P* value <0.05 was considered significant. We ran two multivariate logistic regression analyses. In model 1, we tested the effect of significant variables obtained in the bivariate analysis on the dependent variable: abnormal STS; in model 2, we tested variables with a plausible causal effect on the abnormal STS task. An estimated weight of the independent variables was assessed with exponentiation of B (Exp B) coefficient. Goodness of fit of the regression model was evaluated with the Hosmer–Lemeshow test, and a *P* value <0.05 was considered as poor fit.

## 4. Results

### 4.1. PD Patients with and without Abnormal STS Task

We studied 92 consecutive patients with PD. Two patients were excluded, one had lumbar stenosis, resulting in lower limb weakness, and one had a glioma presenting initially with Parkinsonism. We analyzed 90 patients with PD. There were 20 (22.2%) patients with abnormal STS task (item 3.9, ≥2 points). There were no differences in age and sex distribution between PD patients with and without abnormal STS task. The evolution time of PD did not differ between groups. However, patients with abnormal STS task had a higher modified HY stage (*P*  <  0.001) and MDS-UPDRS-III total score (*P*  <  0.001) and were taking higher doses of LEDD, although this variable did not reach statistical significance (*P* = 0.174) (Table 1). No difference in OH was observed between groups. A higher proportion of perceived lower limbs weakness and allied sensations (*P* = 0.004) was observed among patients with abnormal STS task, and they rated these abnormal sensations greater than PD patients with normal STS task (*P*  <  0.001) (Table 1). The presence of perceived lower limb weakness greatly increased the risk of an abnormal STS task, OR: 11.93 (95% C.I. 1.51–94.32).

Patients with PD and abnormal STS task had a significantly higher composite score for leg bradykinesia (*P*  <  0.001) (Table 1). Leg tremor did not show significance between groups (*P* = 0.405), whereas leg rigidity showed a statistically significant trend (*P* = 0.053). Items for gait, “freezing,” and postural instability were higher in patients with abnormal STS task (*P*  <  0.001, for all variables). A hip extension strength ≤9 kg increased the risk of an abnormal STS task, OR: 4.45 (95% C.I. 1.49–13.23). The lower muscle strength at the hip level was observed in patients with abnormal STS task (Table 1).

In the multivariate analysis, including statistically significant variables from the bivariate analysis as independent (model 1), gait and leg strength (≤9 kg) were included in the final model. In model 2, we included composite leg tremor, leg rigidity, and leg bradykinesia scores as well as leg strength for hip extension (≤9 kg); only the latter two variables showed significance in the final regression model (Table 2). When variables that may explain an abnormal STS task were included as independent (model 2) such as leg bradykinesia, leg rigidity, leg tremor, and muscle strength for hip extension, age, sex, and evolution time of PD; only leg bradykinesia and leg strength (≤9 kg) were included in the final regression model (Table 2).

### 4.2. PD Patients with Abnormal STS Task vs. Controls

We compared 20 patients with PD and abnormal STS task with sex- and age-matched controls. Patients with PD and abnormal STS task endorsed lower limb weakness much more frequently than controls: 95 vs. 21% (P<0.001) and rated their abnormal lower limb sensations as more severe than controls(*P*  <  0.001) (Table 3). Decreased muscle strength at the hip level was detected in patients compared with controls by MRC or quantitative assessment (Table 3).

## 5. Discussion

In this study, we found that patients with PD and moderate-to-severe abnormal STS task (≥2 points in item 3.9 of the MDS-UPDRS-III) have increased complains related to the lower limb weakness and rated such weakness as more severe compared with PD patients with normal STS task. The presence of perceived lower limb weakness increased the risk of an abnormal STS task by 11.93.

Patients with PD and moderate-to-severe abnormal STS task also had higher scores in the Hoehn and Yahr and MDS-UPDRS part III, suggesting that central motor abnormalities, particularly the lower limb bradykinesia and rigidity, may play a role in difficulties rising from a chair. These patients had decreased muscle strength in the lower extremities compared with PD patients with normal STS task and normal controls. This weakness was detected at the hip level by assessing isokinetic muscle contractions, suggesting that decreased torque at the coxofemoral joint may influence the dynamics of the STS task. We found that decreased muscle torque for hip extension (≤9 kg) significantly increased the risk for having an abnormal STS task. However, this difference was not found with hip flexion assessed by dynamometric measures.

The relatively small difference in muscle strength between PD patients with normal or abnormal STS task or normal controls suggests that such decreased muscle strength only partially explains the difficulties in the STS task in most patients with PD and is difficult to detect in routine clinical examination.

**Figure 1:**
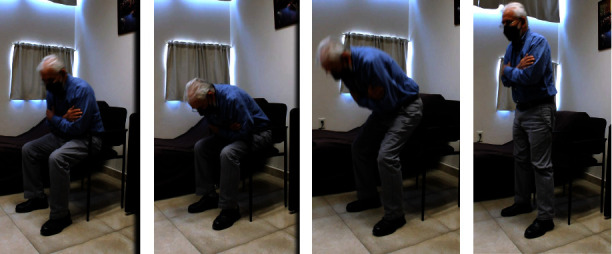
The STS task in Parkinson's disease. (a, b) Increased compensatory trunk flexion, patients tend to sit down near the border of a chair. (c, d) Patients show increased velocity during the rising stage, increasing the forward instability but decreasing backward instability.

Body kinematics have shown that this technique is useful to classify individuals with and without PD after assessing the STS task [20, 21]. Patients with PD use greater preparative strategies compared to normal subjects to stand up by means of increased preparatory hip or trunk flexion with greater forward displacement of the center-of-mass (COM) (Figures 1(a) and 1(b)) [4, 22]. It has been postulated that such preparatory movements are related to decreased muscle strength in the lower extremities, particularly at the hip level [22]. Indeed, decreased hip and knee extensor strength was observed in 10 patients with PD, compared with 10 sex- and age-matched controls in one study [5]. Our study supports this observation. The greater forward body inclination observed in patients with PD as a strategy to achieve the STS task may result in greater backward stability at the expense of increased risk of forward balance loss at the time of movement termination [23].

Usually, patients with PD have shown prolonged STS total times compared with healthy controls [24]. This difference has been attributed to inappropriate peak hip flexion and ankle dorsiflexion torques, prolonged torque production, and difficulty to shift from the flexion to extension direction during the STS task [24]. Patients with PD may take longer to generate the force to achieve the STS task, and they dedicate a larger proportion of time to complete the flexion momentum phase compared with controls [2].

Previous studies have shown that maximal velocity of the COM and limb support determined by the hip height at the instant of peak velocity were predictors of success in the STS task (Figures 1(c) and 1(d)), whereas dynamic stability does not seem to predict such success [6, 25]. Patients with PD redistribute their joint torques in the lower extremities when performing the STS task; this phenomenon seems related to compensation in order to try to gain postural stability [26]. Patients with PD may show increased angular velocity of the trunk in the sagittal plane to complete the STS task, even at the early stages of the disease compared to healthy individuals supporting the notion that appropriate acceleration while performing the STS task is key to achieve it successfully. Patients with PD suffering more postural instability and gait difficulty had shown a slower anteroposterior COM velocity displacement, while performing the STS task by biomechanical analysis [25]. We confirmed those findings as in our study patients with PD, and abnormal STS task had significantly greater scores (worse) in gait, freezing, and postural instability than patients with PD and unaltered STS task.

The pathophysiology of decreased muscle strength in PD is not fully understood. A central mechanism has been postulated as the Parkinsonian features does not fully explain the reduced muscle strength [5]. In our study, we found that bradykinesia and decreased muscle strength were independently related with abnormal STS task; however, the coefficient of correlation (*R*^2^) was low in the final regression model, suggesting that these two variables explain less than 25% of the STS task abnormality, indicating that other mechanisms may play an important role.

Intrinsic abnormalities in skeletal muscles have been detected in patients with PD, as there is higher distribution and larger cross-sectional area of type I muscle fibers, which is considered a compensatory mechanism for heterogenic changes of type II fibers [27]. However, mitochondrial dysfunction with decreased *β*-oxidation in muscles and decreased 12–14 long-chain acylcarnitines during the early stages of PD patients have been reported [28, 29].

Our study has a number of limitations; we were not able to compare males with females. Moreover, we did not carry computed kinematics that would help to determine which aspects of the STS task correlate better with the hip torque. In our study, we did not determine the effect of therapy in the muscle strength and STS task. There is evidence that task-specific training using preparatory audiovisual cues may improve overall dynamic stability in forward and backward balance loss [23]. Audiovisual cues increase hip flexion and knee extension torques, decrease time to complete the STS task, and decrease the time to peak joint torques with increase in peak horizontal and vertical velocities of COM [30]. Another limitation is that we did not assess the presence of recent falls that may be related to abnormal; however, no patient had a history of recent bone fractures or surgery for joint prothesis.

## 6. Concluding Remarks

Abnormalities in the STS task have an important role in mobility in patients with PD. The pathogenesis of such abnormalities is not fully understood; but it seems to implicate dynamic muscle adjustments to control displacement of the COM and appropriate torque transmission to the joints of the lower extremities. Patients with abnormal STS task frequently complain of the lower limb weakness and allied sensation and have decreased muscle strength particularly around the hips. Muscle strength ≤9 kg for hip extension and leg bradykinesia were independently associated with an abnormal STS task; this suggests a role of peripheral and central mechanisms for abnormal chair rising in patients with PD.

## Figures and Tables

**Table 1:** Clinical and demographic features between patients with abnormal and normal sit-to-stand tasks.

	PD patients with abnormal STS task, *n* = 20 (%)	PD patients with normal STS task, *n* = 70 (%)	*P* value
Age at evaluation (years)	70.9 ± 8.1	67.2 ± 10.7	0.160
Sex (male)	11 (55)	40 (57)	0.865
PD time since onset (years)	9.55 ± 9.03	7.02 ± 5.73	0.139
MDS-UPDRS-III total score	47.95 ± 13.77	31.07 ± 15.01	<0.001
Leg rigidity	1.97 ± 0.88	1.45 ± 0.92	0.053
Leg bradykinesia	1.86 ± 0.74	1.09 ± 0.83	<0.001
R Leg bradykinesia	1.55 ± 0.90	1.00 ± 0.95	0.015
L Leg bradykinesia	2.17 ± 0.71	1.23 ± 0.92	<0.001
Gait	3.10 ± 0.64	1.46 ± 0.93	<0.001
Freezing	2.10 ± 1.80	0.40 ± 0.90	<0.001
Postural stability	2.10 ± 0.86	1.33 ± 0.99	<0.001
Leg tremor	0.45 ± 0.77	0.30 ± 0.63	0.405
Modified Hoehn and Yahr stage	4.20 ± 0.76	3.11 ± 1.63	<0.001
LEDD	808.7 ± 618.6	597.2 ± 606.5	0.174
Orthostatic hypotension^*∗*^	8 (40)	18 (25.7)	0.214
Weakness and allied sensations			
Affected patients	19 (95)	43 (61.4)	0.004
Severity by VAS (0–10)	7.35 ± 2.45	4.33 ± 3.64	<0.001
Clinical examination (MRC)			
Right hip flexion	4.55 ± 0.51	4.87 ± 0.34	0.014
Right leg extension	4.80 ± 0.41	4.97 ± 0.17	0.082
Right foot plantar flexion	5.00 ± 0.00	5.00 ± 0.00	—
Left hip flexion	4.55 ± 0.51	4.87 ± 0.36	0.014
Left leg extension	4.70 ± 0.47	4.97 ± 0.17	0.019
Left foot plantar flexion	5.00 ± 0.00	5.00 ± 0.00	—
Quantitative assessments (kg)			
Right hip flexion	12.12 ± 2.80	14.06 ± 2.79	0.290
Left hip flexion	13.24 ± 2.04	14.36 ± 2.83	0.072
Right hip extension	10.57 ± 5.20	12.76 ± 4.49	0.078
Left hip extension	9.83 ± 6.25	12.62 ± 4.35	0.030

LEDD, levodopa equivalent daily dose; MDS-UPDRS-III, Movement Disorders Society Unified Parkinson's Disease Rating Scale part III; MRC, Medical Research Council; VAS, visual analog scale. ^*∗*^OH is defined as drop in the systolic blood pressure ≥20 mmHg or diastolic blood pressure ≥10 mmHg.

**Table 2:** Multivariate analysis to assess the effect of muscle strength and Parkinsonian variables on the sit-to-stand task.

Variables in the final equation	Exp B coefficient	Exp B 95% C.I.	*P* value
Model 1			
Gait	10.394	3.268–33.05	<0.001
Strength ≤9 kg	4.616	1.021–20.881	0.047
Model 2			
Bradykinesia	2.646	1.353–5.174	0.004
Strength ≤9 kg	3.602	1.126–11.517	0.031

Model 1: constant: B-7.630, Exp B: 0.001, *P*  <  0.001. Hosmer–Lemeshow: (*P* = 0.993). Variables not included in the final equation: leg rigidity, leg bradykinesia, freezing, and postural instability. Model 2: constant: B-3.342, Exp B: 0.035, *P*  <  0.001. Hosmer–Lemeshow: (*P* = 0.709). Variables not included in the final equation: leg tremor and leg rigidity. Cox and Snell *R*^2^: 0.173; Nagelkerke *R*^2^: 0.270. The same significant variables were obtained in model 2, when introducing sex, age, and time of evolution of Parkinson's disease as independent variables into the regression model. Method: backward (Wald) for both models.

**Table 3:** Clinical and demographic features between patients with PD and controls.

	PD patients with abnormal STS task, *n* = 20 (%)	Healthy controls, *n* = 52 (%)	*P* value
Age at evaluation (years)	70.9 ± 8.0	68.8 ± 8.9	0.369
Sex (male)	11 (55)	26 (50)	0.704
MDS-UPDRS-III (total score)	47.95 ± 13.77	1.65 ± 3.41	<0.001
Weakness and allied sensations			
Affected patients	19 (95)	11 (21)	<0.001
Severity (0–10)	7.35 ± 2.45	2.00 ± 2.98	<0.001
Clinical examination (MRC)			
Right hip flexion	4.55 ± 0.51	4.90 ± 0.30	0.008
Right leg extension	4.80 ± 0.19	4.96 ± 0.19	0.105
Right foot plantar flexion	5.0 ± 0.0	4.98 ± 0.14	0.539
Left hip flexion	4.55 ± 0.51	4.90 ± 0.30	0.008
Left leg extension	4.70 ± 0.47	4.96 ± 0.19	0.025
Left foot plantar flexion	5.0 ± 0.0	4.98 ± 0.14	0.539
Quantitative assessments (kg)			
Right hip flexion	13.25 ± 2.04	14.38 ± 3.09	0.322
Left hip flexion	12.12 ± 2.80	14.13 ± 3.30	0.108
Right hip extension	10.58 ± 5.20	15.87 ± 7.29	0.006
Left hip extension	9.83 ± 6.25	15.67 ± 7.20	0.003

MDS-UPDRS-III, Movement Disorders Society Unified Parkinson's Disease Rating Scale part III; MRC, Medical Research Council; VAS, visual analog scale.

## Data Availability

The clinical data used to support the findings of this study are available from the corresponding author upon request.

## Additional Points

*Permissions*. The International Parkinson's Disease and Movement Disorders Society provided permission to use the MDS-UPDRS scale for this study.

## References

[1] Jankovic J. (2008). Parkinson’s disease: clinical features and diagnosis. *Journal of Neurology, Neurosurgery & Psychiatry*.

[2] Bishop M., Brunt D., Pathare N., Ko M., Marjama-Lyons J. (2005). Changes in distal muscle timing may contribute to slowness during sit to stand in Parkinsons disease. *Clinical biomechanics*.

[3] Domingues V. L., Pompeu J. E., de Freitas T. B., Polese J., Torriani-Pasin C. (2022). Physical activity level is associated with gait performance and five times sit-to-stand in Parkinson’s disease individuals. *Acta Neurologica Belgica*.

[4] Nikfekr E., Kerr K., Attfield S., Playford E. (2002). Trunk movement in Parkinson’s disease during rising from seated position. *Movement Disorders*.

[5] Inkster L. M., Eng J. J., MacIntyre D. L., Stoessl A. J. (2003). Leg muscle strength is reduced in Parkinson’s disease and relates to the ability to rise from a chair. *Movement Disorders*.

[6] Mak M. K., Yang F., Pai Y. C. (2011). Limb collapse, rather than instability, causes failure in sit-to-stand performance among patients with Parkinson disease. *Physical Therapy*.

[7] Friedman J. H., Abrantes A. M. (2012). Self perceived weakness in Parkinson’s disease. *Parkinsonism & Related Disorders*.

[8] Alonso-Juarez M., Fekete R., Baizabal-Carvallo J. F. (2022). Objective and self-perceived lower limb weakness in Parkinson’s disease. *Therapeutic advances in neurological disorders*.

[9] Koller W., Kase S. (1986). Muscle strength testing in Parkinson’s disease. *European Neurology*.

[10] Nogaki H., Kakinuma S., Morimatsu M. (2001). Muscle weakness in Parkinson’s disease: a follow-up study. *Parkinsonism & Related Disorders*.

[11] Paul S. S., Canning C. G., Sherrington C., Fung V. S. (2012). Reduced muscle strength is the major determinant of reduced leg muscle power in Parkinson’s disease. *Parkinsonism & Related Disorders*.

[12] Stevens-Lapsley J., Kluger B. M., Schenkman M. (2012). Quadriceps muscle weakness, activation deficits, and fatigue with Parkinson disease. *Neurorehabilitation and Neural Repair*.

[13] Ramsey V. K., Miszko T. A., Horvat M. (2004). Muscle activation and force production in Parkinson’s patients during sit to stand transfers. *Clinical biomechanics*.

[14] Hughes A. J., Daniel S. E., Kilford L., Lees A. J. (1992). Accuracy of clinical diagnosis of idiopathic Parkinson’s disease: a clinico-pathological study of 100 cases. *Journal of Neurology, Neurosurgery & Psychiatry*.

[15] Goetz C. G., Poewe W., Rascol O. (2004). *Movement* disorder society task force report on the Hoehn and Yahr staging scale: status and recommendations the *movement* disorder society task force on rating scales for Parkinson’s disease. *Movement Disorders*.

[16] Schade S., Mollenhauer B., Trenkwalder C. (2020). Levodopa equivalent dose conversion factors: an updated proposal including opicapone and safinamide. *Movement disorders clinical practice*.

[17] Metzler M., Duerr S., Granata R., Krismer F., Robertson D., Wenning G. K. (2013). Neurogenic orthostatic hypotension: pathophysiology, evaluation, and management. *Journal of Neurology*.

[18] Paternostro-Sluga T., Grim-Stieger M., Posch M. (2008). Reliability and validity of the Medical Research Council (MRC) scale and a modified scale for testing muscle strength in patients with radial palsy. *Journal of Rehabilitation Medicine*.

[19] Nogaki H., Kakinuma S., Morimatsu M. (1999). Movement velocity dependent muscle strength in Parkinson’s disease. *Acta Neurologica Scandinavica*.

[20] Adamowicz L., Karahanoglu F. I., Cicalo C. (2020). Assessment of sit-to-stand transfers during daily life using an accelerometer on the lower back. *Sensors*.

[21] Wairagkar M., Villeneuve E., King R. (2022). A novel approach for modelling and classifying sit-to-stand kinematics using inertial sensors. *PLoS One*.

[22] Inkster L. M., Eng J. J. (2004). Postural control during a sit-to-stand task in individuals with mild Parkinson’s disease. *Experimental Brain Research*.

[23] Bhatt T., Yang F., Mak M. K., Hui-Chan C. W., Pai Y. C. (2013). Effect of externally cued training on dynamic stability control during the sit-to-stand task in people with Parkinson disease. *Physical Therapy*.

[24] Mak M. K., Hui-Chan C. W. (2002). Switching of movement direction is central to parkinsonian bradykinesia in sit-to-stand. *Movement Disorders*.

[25] Pelicioni P. H. S., Pereira M. P., Lahr J., Rodrigues M. M. L., Gobbi L. T. B. (2020). Biomechanical analysis of sit-to-walk in different Parkinson’s disease subtypes. *Clinical biomechanics*.

[26] Skinner J. W., Lee H. K., Hass C. J. (2021). Redistribution of joint moments and dynamic balance control during sit to stand task in persons with Parkinson’s disease. *Parkinsonism & Related Disorders*.

[27] Kelly N. A., Ford M. P., Standaert D. G. (1985). Novel, high-intensity exercise prescription improves muscle mass, mitochondrial function, and physical capacity in individuals with Parkinson’s disease. *Journal of Applied Physiology*.

[28] Saiki S., Hatano T., Fujimaki M. (2017). Decreased long-chain acylcarnitines from insufficient *β*-oxidation as potential early diagnostic markers for Parkinson’s disease. *Scientific Reports*.

[29] Blin O., Desnuelle C., Rascol O. (1994). Mitochondrial respiratory failure in skeletal muscle from patients with Parkinson’s disease and multiple system atrophy. *Journal of Neurological Sciences*.

[30] Mak M. K., Hui-Chan C. W. (2004). Audiovisual cues can enhance sit-to-stand in patients with Parkinson’s disease. *Movement Disorders*.

